# Systematic Review of Tongxinluo Capsule on the Therapeutic Effect and Hemorheology of Patients with Transient Ischemic Attack

**DOI:** 10.1155/2021/5541768

**Published:** 2021-12-06

**Authors:** Peng Yang, Peng Liu, Ruijin Yang

**Affiliations:** Ganzhou People's Hospital/Ganzhou Hospital, Nanchang University, Ganzhou 341000, China

## Abstract

**Objectives:**

This study aims to systematically evaluate the clinical efficacy of Tongxinluo capsule in the treatment of transient ischemic attack (TIA) and its effect on hemorheology, thereby providing scientific basis for clinical decision making.

**Methods:**

A comprehensive and systematic literature retrieval was conducted in the CNKI, Wanfang database, SinoMed, EMbase, and PubMed to screen the randomized controlled trials (RCTs) of Tongxinluo capsule in the treatment of TIA. The retrieval time was from the inception of each database to September 10, 2020. Endnote X9 was used to screen the literature. Cochrane Collaboration tool for assessing risk of bias was used to evaluate the quality of the included studies. Stata16.0 statistical software was used for meta-analysis.

**Results:**

A total of 12 RCTs were included, involving 946 subjects. (1) The clinical efficacy of the Tongxinluo group was better than that of the control group (RR = 1.19, 95% CI (1.09, 1.30), *P* *≤* 0.001). (2) The hemorheological characteristics of the Tongxinluo group were significantly improved compared with those of the control group (whole blood high shear viscosity: SMD = −1.61, 95% CI (−1.89, −1.34); *P* *≤* 0.001, whole blood low shear viscosity: SMD = −1.06, 95% CI (−1.31, −0.80), *P* *≤* 0.001, fibrinogen: SMD = −1.12, 95% CI (−1.94, −0.29), *P* = 0.008, plasma specific viscosity: SMD = −1.00, 95% CI (−1.69, −0.31), *P* = 0.004, and hematocrit: SMD = −1.47, 95% CI (−2.16, −0.77), *P* *≤* 0.001). (3) There was no significant difference in the incidence of adverse reactions between the Tongxinluo group and control group (RR = 7.76, 95% CI (0.98, 61.28), *P* = 0.052).

**Conclusion:**

Tongxinluo capsule is superior to conventional treatment in improving clinical overall response rate and hemorheological indexes and is relatively safe. Due to the deficiencies of the existing studies, more high-quality studies with rigorous design are required for further verification.

## 1. Introduction

Transient ischemic attack (TIA) is defined as a transient neurological dysfunction caused by focal cerebral, spinal, or retinal ischemia, without acute infarction [1]. TIA is the most important independent risk factor and the most critical warning signal of ischemic stroke [2]. About 1/3 TIA patients are form cerebral infarction, and most of them occur within 7 days [3]. Active and effective treatment of TIA can prevent stroke to the greatest extent, which is the key to reduce the morbidity and mortality of cerebrovascular diseases. The pathogenesis of TIA has not been fully determined yet. Emerging evidence has indicated that TIA is often associated with the alternation of hemorheology [4]. The increased blood viscosity causes microcirculation disturbance in patients with cerebrovascular diseases and then induces transient cerebral ischemia, so abnormal hemorheology is an important inducer of TIA [5]. Hemorheology is a science that studies the rheological properties, changing rules and medical applications of blood and tangible components [6], the core of which is the change of its indexes. It is a crucial clue of etiology, prevention, diagnosis, treatment observation, and monitoring of many diseases, which represents an indispensable tool in clinical research [7, 8].

TIA is classified as a type of stroke in traditional Chinese medical science, which originates from deficiency of Qi and arises from blood stasis [9, 10]. At present, traditional Chinese medicine (TCM) mostly aims at promoting blood circulation and removing blood stasis to prevent and treat TIA. The drugs of promoting blood circulation and removing blood stasis are essential for the treatment of TIA [11], which can eliminate the etiology and pathology of TIA caused by blood stasis, thus preventing and controlling TIA from various aspects. With the deepening of the research on biological indicators and TCM syndromes, the correlation between blood stasis syndrome and abnormal hemorheology has been confirmed [12, 13]. Hemorheology index is indispensable for the diagnosis, clinical efficacy evaluation, and drug research of blood stasis syndrome [14]. Tongxinluo (TXL) capsule is a traditional Chinese medicine compound preparation approved by the State Food and Drug Administration of China in 2010. TXL possesses the effects of invigorating Qi, promoting blood circulation, removing blood stasis, and relieving pain, which has achieved significant therapeutic effect on cardiovascular and cerebrovascular diseases [15, 16]. The clinical evidence has demonstrated that TXL capsule can improve the function of vascular endothelial cells, reduce blood viscosity, regulate blood lipid, improve hemorheology, and stabilize atherosclerotic plaques, thereby retarding and alleviating vasospasm of TIA [16].

At present, accumulating clinical trials have reported the beneficial effect of TXL on TIA patients, including improving the therapeutic effect and hemorheological indexes of TIA patients. However, the quality and efficacy of these trials have not been confirmed. Hence, it is necessary to conduct a systematic review on the role of TXL capsule in the treatment of TIA, so as to confer more evidence for the follow-up studies and guidelines.

## 2. Materials and Methods

### 2.1. Inclusion Criteria

#### 2.1.1. Type of Studies

RCTs published at home and abroad in blinding or not were included. The languages were restricted to Chinese and English.

#### 2.1.2. Subject of Studies

The included patients met the diagnostic criteria of TIA in *Diagnostic Essentials of Various Cerebrovascular Diseases* in 1995 [17] or met the latest definition of TIA proposed by the American Heart Association/American Stroke Association in 2009 [1]. There were no restrictions on race, gender, age, and degree of illness.

#### 2.1.3. Intervention Measures

The experimental group was treated with TXL capsule alone or combined with aspirin, while the control group was treated with aspirin. Both the two groups received conventional treatment measures, including improving blood circulation, nourishing nerve support, and expanding blood vessels.

#### 2.1.4. Outcome Measures

① Clinical efficacy; ② hemorheological indexes: whole blood low shear viscosity, whole blood high shear viscosity, plasma specific viscosity, fibrinogen, and hematocrit; and ③ adverse drug reactions.

#### 2.1.5. Exclusion Criteria

① Animal experiment; ② review and systematic evaluation; ③ experience report of famous doctors; ④ case report; ⑤ repetitive publications; ⑥ literature with similar data published by different authors; and ⑦ literature that cannot be traced back to experimental data.

### 2.2. Retrieval Strategies

RCTs about TXL capsule in the treatment of TIA were retrieved in the CNKI, Wanfang database, SinoMed, EMbase, and PubMed, from the inception of each database to September 10, 2020. The full-text retrieval was conducted by combining subject words and free words, and the references included in these studies were retrospected. The key words in Chinese were “Tongxinluo,” “Tongxinluo capsule” and “transient ischemic attack.” The key words in English are “Tongxinluo,” “transient ischemic attack,” and “TIA.”

### 2.3. Data Extraction

The data independently extracted by the two reviewers included (1) basic information: author, title, and year of publication; (2) methodological information: sample size and study design; (3) participants: age, gender, and diagnostic criteria; (4) interventions of the treatment and control groups; (5) outcome measures; and (6) adverse events. Any disagreement between reviewers was settled by consensus or the third reviewer.

### 2.4. Quality Assessments

The two reviewers evaluated the methodological quality of the included studies according to the Cochrane Collaboration tool for assessing risk of bias, including (1) random sequence generation; (2) allocation concealment; (3) blinding of researchers and participants; (4) blinding of outcome assessment; (5) integrity of outcome data; (6) selective outcome reporting; and (7) other bias. The included studies were assessed as high, low, and unclear risk of bias.

### 2.5. Statistical Analysis

Stata16.0 statistical software was used for data analysis. Binomial data were represented by risk ratio (RR), and continuous data were represented by weighted mean difference (WMD) or standardized mean difference (SMD), with corresponding 95% confidence interval (CI). The chi-squared (*χ*^2^) test and *I*^2^ test were used to evaluate the heterogeneity of included studies. If there was no significant heterogeneity (*P* > 0.1 or *I*^2^ < 50%), a fixed-effect model was used; otherwise, a random-effect model was used. A funnel plot was used to assess publication bias. Sensitivity analysis was performed by excluding any of the included trials, so as to evaluate the impact of each study on the pooled results.

## 3. Results

### 3.1. Results of Literature Retrieval

**Figure 1:**
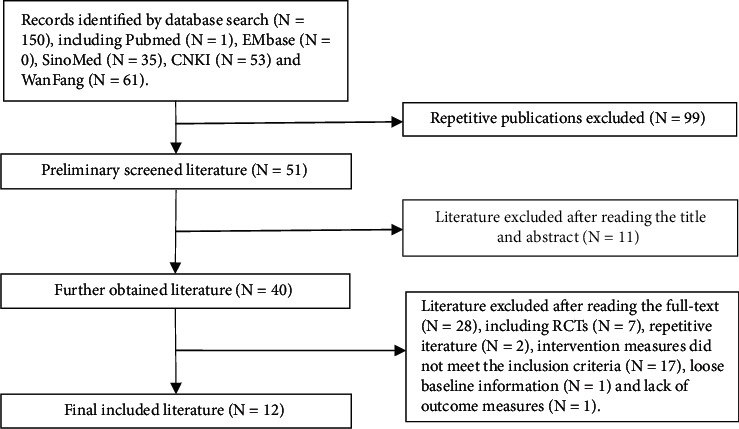
Steps of literature screening.

Based on the retrieval strategies, 152 related studies were preliminarily obtained. Among them, 100 repetitive studies were excluded, and 11 studies were excluded after reading the title and abstract. The remaining 41 studies were independently reviewed by two reviewers. After full-text reading, 29 studies that did not meet the inclusion criteria were excluded. Finally, 12 RCTs [18–29] were included, all of which were Chinese literature. The specific screening steps are shown in Figure 1.

### 3.2. Basic Information of the Included Literature

All the 12 RCTs were single-center studies published from 2005 to 2018. All cases were from China. A total of 946 cases were included, including 501 cases in the experimental group and 445 cases in the control group. The main outcome was clearly reported in 12 RCTs, including 3 RCTs on whole blood high shear viscosity, 3 RCTs on whole blood low shear viscosity, 3 RCTs on fibrinogen concentration, 4 RCTs on plasma specific viscosity, and 3 RCTs on hematocrit. The details are summarized in Table 1.

### 3.3. Quality Assessment of the Included Literature

**Figure 2:**
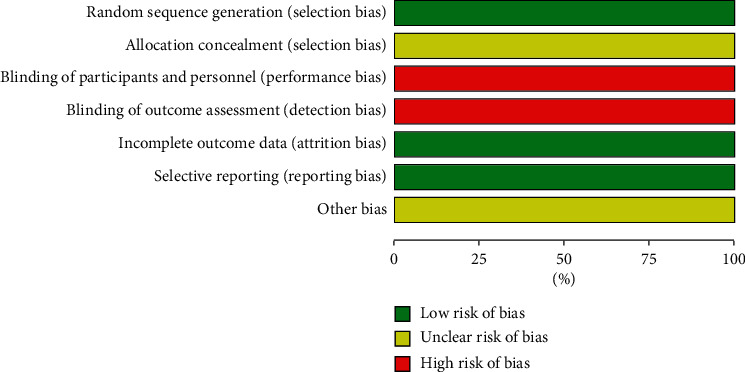
Bar graph of bias risk.

**Figure 3:**
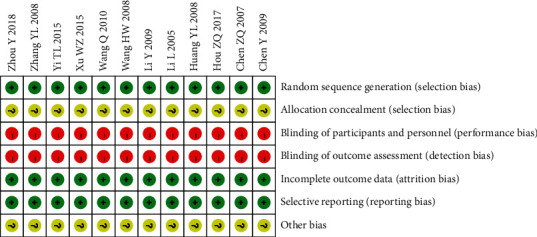
Risk of bias summary.

The included literature was assessed with the Cochrane Collaboration tool, including 7 items. For random sequence generation, 1 study mentioned the random sequence generated by random number table method, and the other 11 studies did not explain the specific random method and merely simply mentioned random. For random allocation concealment, 12 studies did not mention it. For blinding of researchers, participants, and outcome assessment, all 12 studies were not blinding. For integrity of outcome data, there was no case shedding or incomplete outcome data reporting. The level of other bias of the 12 studies was unclear. The risk of bias is shown in Figures 2 and 3.

### 3.4. Meta-Analysis Results

#### 3.4.1. Overall Response Rate

**Figure 4:**
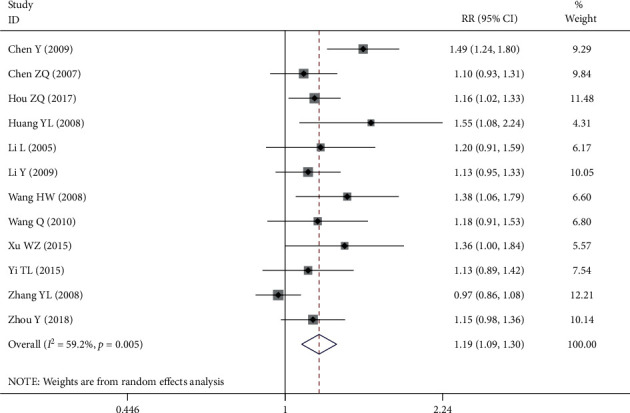
Forest plot of overall response rate.

All 12 RCTs reported the overall response rate. There was statistical heterogeneity among the studies (*P* = 0.005, *I*^2^ = 59.2%), so the random-effect model was used. Meta-analysis showed that the overall response rate of the experimental group was significantly higher than that of the control group (RR = 1.19, 95% CI (1.09, 1.30), *P* *≤* 0.001), as shown in Figure 4.

#### 3.4.2. Sensitivity Analysis

**Figure 5:**
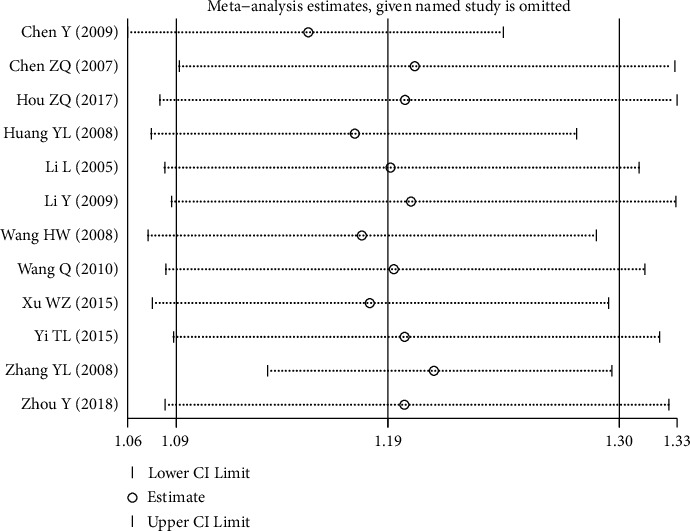
Sensitivity analysis.

Sensitivity analysis was conducted because of the heterogeneity among the included studies.  Figure 5 showed that the two studies by Chen and Zheng [18] and Zhang [28] were the main sources of heterogeneity. After excluding the study by Chen Yan in 2009, there was no significant heterogeneity among the remaining 11 studies (*P* = 0.071, *I*^2^ = 41.8%). The fixed-effect model was used for meta-analysis, and the results showed that the overall response rate of the experimental group was significantly higher than that of the control group (RR = 1.18, 95% CI (1.10, 1.26), *P* *≤* 0.001). After excluding the study by Zhang Yanli in 2008, there was no significant heterogeneity among the remaining 11 studies (*P* = 0.278, *I*^2^ = 17.3%). The fixed-effect model was used for meta-analysis, and the results showed that the overall response rate of the experimental group was significantly higher than that of the control group (RR = 1.24, 95% CI (1.17, 1.33), *P* *≤* 0.001). Each study was excluded one by one, and the meta-analysis was conducted again. The results showed that the overall response rate of the experimental group was still higher than that of the control group. In conclusion, the results of meta-analysis of TXL capsule in the treatment of TIA were reliable, with high confidence level.

#### 3.4.3. Whole Blood High Shear Viscosity

**Figure 6:**
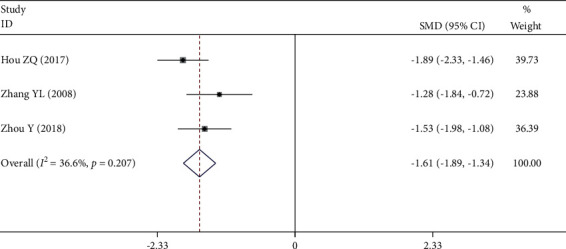
Forest plot of whole blood high shear viscosity.

Three [20, 28, 29] of the 12 RCTs reported whole blood high shear viscosity. There was no significant statistical heterogeneity among these studies (*P* = 0.207, *I*^2^ = 36.6%), so the fixed-effect model was used. Meta-analysis showed that the decrease of whole blood high shear viscosity in the experimental group was significantly lower than that in the control group (SMD = −1.61, 95% CI (−1.89, −1.34), *P* *≤* 0.001), as shown in Figure 6.

#### 3.4.4. Whole Blood Low Shear Viscosity

**Figure 7:**
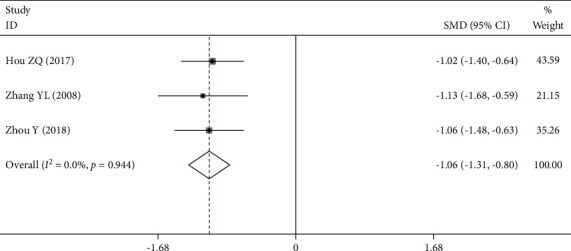
Forest plot of whole blood low shear viscosity.

Three [20, 28, 29] of the 12 RCTs reported whole blood low shear viscosity. There was no significant statistical heterogeneity among the studies (*P* = 0.944, *I*^2^ = 0.0%), so the fixed effect model was used. Meta-analysis showed that the decrease of whole blood low shear viscosity in the experimental group was significantly lower than that in the control group (SMD = −1.06, 95% CI (−1.31, −0.80), *P* *≤* 0.001), as shown in Figure 7.

#### 3.4.5. Fibrinogen

**Figure 8:**
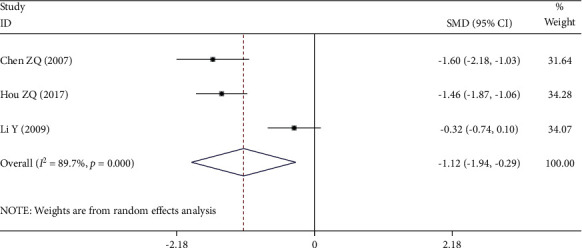
Forest plot of fibrinogen.

Three [19, 20, 23] of the 12 RCTs reported fibrinogen. There was a significant statistical heterogeneity among the studies (*P* *≤* 0.001, *I*^2^ = 89.7%), so the random-effect model was used. Meta-analysis showed that the decrease of fibrinogen in the experimental group was significantly lower than that in the control group (SMD = −1.12, 95% CI (−1.94, −0.29), *P* = 0.008), as shown in Figure 8.

#### 3.4.6. Plasma Specific Viscosity

**Figure 9:**
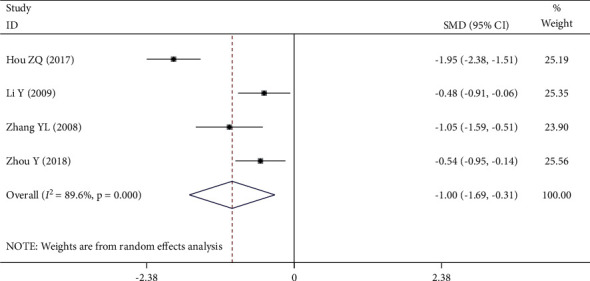
Forest plot of plasma specific viscosity.

Four [20, 23, 28, 29] of the 12 RCTs reported plasma specific viscosity. There was significant statistical heterogeneity among the studies (*P* *≤* 0.001, *I*^2^ = 89.6%), so the random-effect model was used. Meta-analysis showed that the decrease of plasma specific viscosity in the experimental group was significantly lower than that in the control group (SMD = −1.00, 95% CI (−1.69, −0.31), *P* = 0.004), as shown in Figure 9.

#### 3.4.7. Hematocrit

**Figure 10:**
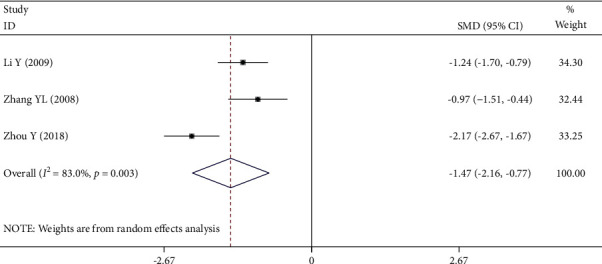
Forest plot of hematocrit.

Three [23, 28, 29] of the 12 RCTs reported hematocrit. There was significant statistical heterogeneity among the studies (*P* = 0.003, *I*^2^ = 83%), so the random-effect model was used. Meta-analysis showed that the decrease of hematocrit in the experimental group was significantly lower than that in the control group (SMD = −1.47, 95%CI (−2.16, −0.77); *P* *≤* 0.001), as shown in Figure 10.

### 3.5. Incidence of Adverse Reactions

**Figure 11:**
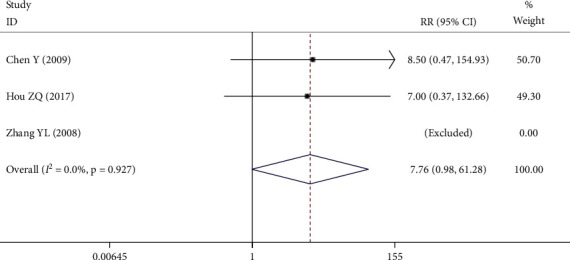
Forest plot of adverse reaction.

Among the 12 RCTs, 3 RCTs reported adverse reactions: 1 RCT [18] reported that 4 patients in the experimental group had mild gastric discomfort and nausea, and the symptoms were relieved after symptomatic treatment after meals; 1 RCT [20] reported that 3 patients in the experimental group had gastrointestinal discomfort and 4 patients in the control group had gastrointestinal discomfort; 1 RCT [28] reported that there were no adverse reactions in the experimental group and the control group. Since there was no statistical heterogeneity among these studies (*P* = 0.927, *I*^2^ = 0.0%), and the fixed-effect model was used for meta-analysis. There was no statistical difference in the incidence of adverse reactions between the two groups (RR = 7.76, 95% CI (0.98, 61.28), *P* = 0.052), as shown in Figure 11.

### 3.6. Publication Bias Analysis

**Figure 12:**
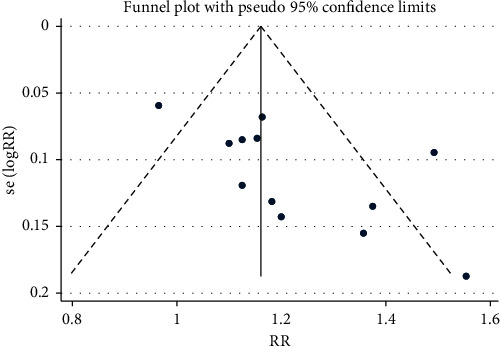
Funnel plot of overall response rate.

A funnel plot was drawn to examine whether there was publication bias in this study, with the overall response rate as the index. The RR value of the included studies was taken as the abscissa, and the reciprocal of the logarithm standard error SE (log[RR]) of RR value was taken as the ordinate. The clinical overall response rate of TXL capsule in the treatment of TIA was analyzed by the funnel plot. Some studies were scattered outside the confidence interval, and the distribution of each point was not completely symmetrical, indicating the small publication bias in the included studies, as shown in Figure 12.

## 4. Discussion

Due to the aging of population and unhealthy lifestyle, the incidence rate of TIA in China is mounting year by year [30], which has aroused increasing attention in the society. At present, antiplatelet drugs are commonly used to alleviate blood hypercoagulability, thereby preventing and treating ischemic stroke or TIA [31]. Aspirin is the most well-documented antiplatelet drug recommended by the secondary prevention guidelines of China and the United States [32, 33]. Although many patients have been taking aspirin to inhibit platelet aggregation and prevent the formation of related embolism, TIA frequency is still high [34]. Hence, complementary and/or alternative medicine is being explored around the world. In recent years, TCM shows good efficacy in TIA. According to the theory of TCM, the main syndrome type of TIA is “blood stasis.” For blood stasis, the treatment principle is to promote blood circulation and eliminate blood stasis. The current research [35] has demonstrated that most of the drugs for activating blood circulation and removing stasis possess the pharmacological actions of improving hemorheology abnormality and microcirculation and preventing thrombosis and atherosclerosis, which have the unique advantages in reducing the morbidity and mortality of ischemic cerebrovascular diseases.

TXL capsule is composed of various active ingredients, including ginseng, leech, scorpion, *Paeonia lactiflora*, cicada slough, woodlouse bug, centipede, and sandalwood. It is a Chinese patent medicine that can improve blood circulation and remove blood stasis. This study found that the overall response rate of patients treated with conventional Western medicine plus Tongxinluo capsule was significantly higher than that of the control patients, and it also had obvious advantages in improving hemorheology indexes. Modern clinical research has revealed that ginseng in TXL capsule has the effect of replenishing Qi; leech can act on fibrinogen, thus exerting anticoagulant and antithrombotic effects [36]; woodlouse bug has obvious anticoagulant and antithrombotic effects, and its total alkaloids can enhance hypoxia tolerance of brain [37, 38]; scorpion and centipede can play a role in dredging meridians. The combination of these ingredients can improve the function of vascular endothelium, relieve vasospasm, promote blood circulation, and inhibit platelet aggregation and thrombosis.

At present, there is no systematic review on the role of TXL capsule in the efficacy and hemorheology of TIA patients. An existing systematic review [39] has suggested that TXL capsule can improve the overall response rate, reduce the viscosity of blood and plasma, and improve the indexes of hemorheology in patients with coronary heart disease. Although coronary heart disease and TIA are a vascular category, they belong to different diseases. Therefore, the study of TXL capsule on the therapeutic effect of TIA and hemorheology has certain innovative significance. This study fills the relevant research gap.

The limitations of this study are as follows: ① the level of domestic clinical evidence is low and lacks high-quality RCTs. ② Some of the outcome indexes have publication bias; some gray literature are not available, and due to the limitation of research methods, this study can only evaluate relevant indexes but cannot exclude potential publication bias. ③ Influence of language and region: although the literature retrieval of this study is not limited to languages, the 12 included RCTs are all in Chinese; it may be that TXL capsule is the Chinese patent medicine, so there are few clinical studies abroad.

In conclusion, we systematically evaluated the effect of TXL capsule on the treatment of TIA and proved that the addition of TXL capsule on the basis of conventional medication was beneficial to the treatment of TIA, which significantly improved the clinical therapeutic effect and hemorheological characteristics, reduced blood viscosity, and promoted microcirculation. Due to the limited quality of included studies, more prospective RCTs with high-quality and large samples are needed for further verification.

## Figures and Tables

**Table 1:** Characteristics of the literature.

Study ID	Sample size	Female		Median age		Intervention		Treatment course	Outcomes
	I/C	I	C	I	C	I	C		
Chen Y, 2009	71/67	44/27	43/24	58.1 ± 8.9	59.6 ± 9.6	TXL + asprin + CT	Asprin + CT	4 weeks	①
Chen ZQ, 2007	35/28	26/19	15/13	NA		TXL + asprin + CT	Asprin + CT	30 days	①②
Hou ZQ, 2017	60/60	36/24	35/25	60.3 ± 4.6	61.5 ± 5.8	TXL + asprin + CT	Asprin + CT	1 month	①②③④⑤
Huan YL, 2008	35/30	20/15	18/12	59.2 ± 10.3	58.1 ± 10.3	TXL + asprin + CT	Asprin + CT	1 month	①
Li L, 2005	30/20	21/9	9/11	62.2 ± 10.5	61.7 ± 10.4	TXL + asprin + CT	Asprin + CT	10 days	①
LI Y, 2009	50/40	30/20	28/12	59.9 ± 9.3/	57.6 ± 8.4	TXL + asprin + CT	Asprin + CT	4 weeks	①②③⑥
Wang HW, 2008	60/30	NA		NA		TXL + asprin + CT	Asprin + CT	15 days	①
Wang Q, 2010	30/30	18/12	16/14	NA		TXL + asprin + CT	Asprin + CT	15 days	①
Xu WZ, 2015	20/20	12/8	11/9	63 ± 7.2	62 ± 6.7	TXL + asprin + CT	Asprin + CT	1 month	①
Yi TL, 2015	31/31	17/14	18/13	62.1 ± 7.8	64.2 ± 8.1	TXL + asprin + CT	Asprin + CT	14 days	①
Zhang YL, 2008	30/30	16/14	13/17	62.76 ± 7.49	61.73 ± 7.54	TXL + CT	Asprin + CT	14 days	①③④⑤⑥
Zhou Y, 2018	49/49	NA		56.71 ± 4.18	57.38 ± 4.29	TXL + asprin + CT	Asprin + CT	14 days	①③④⑤⑥

*Note*. I/C: intervention group and conventional treatment; ①efficient; ②fibrinogen; ③plasma specific viscosity; ④whole blood high shear viscosity; ⑤whole blood low shear viscosity; and ⑥plasma specific viscosity.
